# Complementary and alternative medicine use among older Australian women - a qualitative analysis

**DOI:** 10.1186/1472-6882-12-34

**Published:** 2012-04-04

**Authors:** Deirdre McLaughlin, Chi-Wai Lui, Jon Adams

**Affiliations:** 1 School of Population Health, The University of Queensland, Herston Road, Herston, Queensland 4006, Australia; 2 Faculty of Nursing Midwifery and Health, University of Technology Sydney, Sydney, New South Wales, Australia

**Keywords:** CAM use, Women, Urban, Rural

## Abstract

**Background:**

The use of complementary and alternative medicines (CAM) among older adults is an emerging health issue, however little is known about older people's experiences of using CAM and the cultural, geographical and other determinants of CAM use in this population. This study used qualitative methods to explore older women's views of CAM and reasons for their use of CAM. Participants for the project were drawn from the Australian Longitudinal Study on Women's Health (ALSWH) 1921-1926 birth cohort. Women who responded positively to a question about CAM use in Survey 5 (2008) of the ALSWH were invited to participate in the study. A total of 13 rural and 12 urban women aged between 83 and 88 years agreed to be interviewed.

**Results:**

The women expressed a range of views on CAM which fell into three broad themes: "push" factors such as dissatisfaction with conventional health services, "pull" factors which emphasised the positive aspects of choice and self-care in health matters, and barriers to CAM use. Overall, the "push' factors did not play a major role in the decision to use CAM, rather this was driven by "pull" factors related to health care self-responsibility and being able to source positive information about types of CAM. A number of barriers were identified such as access difficulties associated with increased age, limited mobility and restricted transport options, as well as financial constraints.

**Conclusions:**

CAM use among older women was unlikely to be influenced by aspects of conventional health care ("push factors"), but rather was reflective of the personal beliefs of the women and members of their close social networks ("pull factors"). While it was also apparent that there were differences between the rural and urban women in their use of CAM, the reasons for this were mainly due to the difficulties inherent in accessing certain types of CAM in rural areas.

## Background

The use of complementary and alternative medicines (CAM) among older adults is an emerging health issue [1]. A recent review has suggested that the increasing practice of CAM use among older people may be the result of several interacting trends. For example, the ageing of the global population has resulted in a concomitant focus on compression of morbidity. Additionally, increased consumer participation in health care and the rise of the anti-ageing movement has encouraged the advancement of technologies to slow or reverse the biological process of ageing [2]. However, we know very little about older peoples' experiences of using CAM and there has been criticism of the lack of gerontological perspectives within CAM research. In particular there has been a lack of attention to cultural, geographical and other determinants of CAM use in this population [1].

Despite the increasing prevalence of CAM use in older adults, a common finding in the literature is that older adults report lower use of CAM than other age groups [3-5]. This finding is paradoxical, because research consistently demonstrates that higher levels of chronic conditions and other indicators of poor health are associated with an increase in the use of CAM [6] and older adults generally bear the greatest burden of poor health. The processes which play a role in shaping older adults' use of CAM have been explored in a number of studies which have emphasised the importance of 'push' and 'pull' factors in an older person's decision [7]. 'Push' factors focus on the negative aspects of conventional medicine and include dissatisfaction with conventional health services [8,9], especially for those older people who have not experienced successful control of the symptoms of long-term chronic conditions [10]. The positive 'pull' factors highlight the perceived positive aspects of CAM and have been emphasised in other studies, which suggest that many older people use CAM because it is more consistent with their personal values or because it offers them the opportunity to use their own judgement regarding their health and health care [11,12].

There is evidence that CAM is more commonly utilised by older adults living in rural or non-urban environments [13-15]. This may reflect poorer access to conventional health care services in those areas and may contribute to the 'push' away from conventional services to CAM.

Psychosocial and cultural differences ('pull' factors) may also exist which encourage greater use of CAM among rural residents. Several researchers have suggested that traditional rural values such as self-reliance, individualism and a reluctance to seek medical care unless seriously impaired by health problems may make rural residents hesitant to seek care from conventional health care services [16]. For older people, the most commonly reported source of information and introduction to CAM is word of mouth and information provided by relatives, friends, acquaintances and colleagues [17,18]. Some studies have suggested that informal communications and networks may be particularly strong in rural communities and this may explain higher CAM use in these settings [13,19].

However, there is currently little qualitative research examining older people's rationales for CAM use and how this is influenced by 'push' and 'pull' factors. The aim of this study is to explore the perspectives of older women on CAM use and how this is influenced by geographical location.

## Methods

### Study design

This project is a nested substudy of the Australian Longitudinal Study on Women's Health (ALSWH). The ALSWH is a survey of the health and well-being of three cohorts of women who were aged 18-23 years (1973-1978 birth cohort), 45-50 years (1946-1951 birth cohort) and 70-75 years (1921-1926 birth cohort) when recruited in 1996. Women were selected from the Australian national health insurance database (Medicare), which includes all citizens and permanent residents. Stratified random sampling was used with intentional over-sampling of women from rural and remote areas. The project uses mailed questionnaires to collect self-report data on health and related variables every three years. Details of the cohorts and recruitment methods have been described elsewhere [20].

Participants for this project were drawn from the 1921-1926 birth cohort. 70 older women who responded positively to a question about CAM use in Survey 5 (2008) of the ALSWH and who lived in the south eastern region of Queensland (an Australian State) were sent a mailed invitation to participate in the study (35 rural and 35 urban). A total of 13 rural and 12 urban women aged between 83 and 88 years agreed to participate. Face to face interviews were selected as the most appropriate data collection method to minimise the burden of participation on these older women.

Participation was voluntary and both the ALSWH main study and the nested substudy were approved by the University of Newcastle and University of Queensland Human Research Ethics Committees. All participants provided written consent prior to participation in the interview. Participants were informed that they could stop the interview at any time and decline to answer questions without giving a reason. They could also request that any comments be erased from the recording. In accordance with ethical requirements, all participants were offered the option of reviewing and amending the transcript.

### Data collection

All interviews were conducted by a single interviewer (DM) and followed an interview guide drafted by two researchers (DM and JA). The guide was developed from previous literature and the central research question of the study: 'What factors influence older women's use of CAM and how do these differ according to geographical location?' Key areas that were explored from the central question included history of CAM use, motivations or reasons for using CAM, experiences of using CAM and sources of information about CAM. Because of the age of the women, interviews were conducted in their homes. Interviews lasted from 45 to 120 minutes. Each interview was digitally recorded and then transcribed to a word file. Data collection continued beyond thematic saturation because researchers felt it was important to acknowledge the viewpoints of all the older women who had volunteered.

### Data analysis

Interviews were subjected to an inductive thematic analysis, utilising open, axial and selective coding done concurrently with the qualitative fieldwork process. This sequential approach allows a shaping of the ongoing data collection which has been described as a crucial element in producing high quality data [21]. The resultant themes were mapped to construct a schema of the "push" and "pull" factors identified as impacting on CAM use. As data collection proceeded this schema was further tested and negative cases sought. This resulted in the modification of the schema to include barriers to CAM. Each interview was coded by two researchers (DM and JA) with differences being resolved by mutual agreement.

## Results

The mean age of the women was 84 years (SD = 1.4). Demographic and health characteristics of the women by area of residence are shown in Table 1.

**Table 1:** Demographic and health characteristics of participants

	Rural	Urban
Characteristics	n = 13	n = 12
	n	n
Marital Status		
Married	2	1
Widowed	8	10
Divorced	2	
Never Married	1	1
Manage on Income		
No difficulty	11	11
Some difficulty	2	1
Education*		
Lower	4	4
Middle	4	3
Higher	3	6
Health		
Very Good	1	4
Good	6	7
Fair	6	1
Chronic conditions		
Arthritis	1	3
Osteoporosis	3	4
Macular Degeneration	4	4
Glaucoma	3	1
Cataract	3	6
Heart Disease	3	2
Hypertension	8	7
Skin Cancer	4	4

*Lower (no school or some primary school), Middle (some high school), Higher (trade or tertiary qualification)

The themes that were identified from the transcripts were organised into: (1) "push" factors, such as conventional health services and their impact on CAM use; (2) "pull" factors such as self-responsibility for good health and sources of information about CAM; and (3) barriers to CAM use.

### 'Push' factors

One area that has received some previous research attention is the notion that older women may experience a "push" away from conventional health services because of the difficulties in accessing some of these services and because of the non-participatory role expected of older patients [10]. Despite the widely acknowledged difficulty in accessing health services in regional areas, rural women in this study did not consider that these difficulties influenced their decisions to use CAM. As this woman commented *"I would take my vitamins no matter what"*. Nor did the opinions of their doctors influence their choice of CAM.

"I tell her (doctor) what I'm taking and she looks at it and says it won't hurt me. If she ever said anything, I'd just go somewhere else."(Rural)

Urban women, on the other hand, spoke of the ease with which they could access a wide range of conventional health care services however, they mirrored the rural women's comments that their ability to access health services did not influence their CAM use, nor the type of CAM they utilised. The majority of both urban and rural women told their doctors about their CAM consumption and while one urban woman suggested *"she (the doctor) thought it was a waste of money," *none reported that their practitioner had attempted to influence their decision to use CAM.

While none of the women specifically identified dissatisfaction with, or difficulty in accessing, health services as a reason for their CAM use, the rural women did highlight a number of often frustrating gaps in accessing conventional health services in rural areas. For example, older rural women are often unable to drive the longer distances required to access health services, particularly when these are located in larger towns or cities. One woman spoke of the anxiety she experienced when she needed to see her medical specialist daily for three days and how this arrangement was made without consultation or thought regarding her ability to manage.

"We had to get into the city by 8:00 and that meant leaving home at 4:00 so we could find our way to the surgery. Then he wanted us to come back at the same time for the next two days, so he could keep putting the drops in my eyes. He didn't ask how we were going to manage."(Rural)

Similar difficulties with transport were also raised in relation to accessing CAM and these are discussed in a subsequent section of this paper.

### 'Pull' factors

The women identified a number of positive "pull" factors which influenced their CAM use, including a desire to take responsibility for their own health and well-being and to make their own healthcare choices. The use of CAM to maintain good health was strongly endorsed by both urban and rural women.

"It (supplement) keeps my joints free and I can move around more easily. Do more gardening" (Urban)

"I've been going to a naturopath for years. Took all my kids too. I think it's better to prevent illness than to allow yourself to get sick and then have to take all those (conventional) medications to try and get better" (Rural)

A number of women expanded this theme, including the value of good nutrition, as well as the importance to being proactive in using CAM to maintain good health, as the following quotes illustrate.

"I grow my own vegetables, I eat well and I take vitamins and supplements. I look after myself and only go to the doctor when I really feel under the weather."(Urban)

"I'm a great believer in holistic medicine. If you go to the GP (general medical practitioner), the only thing they do is give you antibiotics. I've been taking these supplements (from an holistic medical practitioner) for two years and I think I would be dead if not for him."(Rural)

The self-sufficiency that is inherent in this older cohort of women, now in their late eighties, extends to their use of CAM. Regardless of where they lived, most of the women took great pride in highlighting their ability to manage their own health needs. Many of the women placed great reliance on old-style natural products and remedies, some learned from their own mothers. The women's comments illustrated that they were active and thoughtful consumers of CAM and recognised that CAM takes many forms, as is evident from the following descriptions.

"We've always looked after ourselves you know. We have always relied on natural remedies first - things that I learned from my mother. She always said the cod liver oil was the only thing for bad chests."(Rural)

"I can't afford to use many bought things (CAM), so I use the old-fashioned remedies. You know, aloe vera for burns and stuff like that. I think they're better than going to the doctor at the drop of a hat."(Urban)

Both the rural and the urban women described receiving information about CAM from a range of sources that were consistent regardless of area of residence. CAM practitioners in particular, were often sought because of recommendation from friends, family and other members of the women's social networks. For instance, this rural woman commented that *"one of my friends took me to a Bowen therapy man." *While another said *"she (a friend) told me of this wonderful chiropractor that she had seen." *The urban women shared similar experiences, for instance, *"I sat with a lady and had a chat with her and she told me about a wonderful naturopath in (suburb)." *Other sources of information that were utilised, particularly in selecting CAM medications, included health food stores (especially those that were co-located with a CAM practitioner), community groups, advertisements (all types of media, but most frequently mentioned were magazines). Pharmacies were mentioned by a number of both urban and rural women as credible sources of information about CAM and also of access to practitioners. In particular, naturopaths and acupuncturists were identified by several of the women as being frequently collocated with pharmacies. This collocation provides an opportunity for women to experience types of CAM that they may not have previously accessed, as this woman describes,

"One day I saw this notice up outside a chemist shop, 'naturopath' and I thought that's good. She was a very nice person and I thought I'll try this out." (Rural)

The women also relied on advice from pharmacists and their staff when purchasing over the counter CAM as this quote illustrates,

"Sometimes chemists are putting something in front of you saying, take this, it will help."(Urban)

In rural areas, the role of country women's groups was cited by the women as being not only a source of social networking opportunities, but also provided a forum in which the women could discuss a wide range of health care experiences. A number of these groups introduced guest speakers who spoke about a wide range of health services and health care, including a naturopath, and several other CAM therapists. As one woman from a remote rural area explained,

"As I say, going to women's groups they often have a speaker from some aspect of health or something and you learn there. That's where I heard about (a naturopath), she gave a talk." (Rural)

This type of information dissemination did not occur in the urban interviews, where the women spoke most frequently about sourcing information from friends, neighbours and, on occasion, the media.

"I read about this in that magazine and thought it looked good. My friend was taking it and she said it certainly upped her energy."(Urban)

When the women were probed about the credibility of their information sources, they placed most reliance on closely connected others, such as family and friends. This is illustrated in the comments of this urban woman,

"My son recommends the things I should take: He's got a Bachelor of Applied Science. He's not an idiot."

### Barriers to CAM use

There were differences in the type of CAM that was available to the women according to their area of residence: rural women were more likely to experience barriers in accessing CAM practitioners and were more restricted in the types of practitioners who were available than women living in cities. However, both CAM practitioners and the rural women were resourceful in overcoming this type of barrier as this woman's description illustrates.

"(The acupuncturist) comes up every month and (my friend) she also has a cottage... it's all furnished, but nobody in it. And that's where he comes every month."(Rural)

Others were prepared to make regular trips to the city to access a practitioner,

"My nephew drives me down (to the city)......it's a long way and it's a long day."(Rural)

These trips however were costly, not only in terms of time, but also in financial resources, as this woman commented,

"Well transport now... It costs you know, anything up to (AUD) $400 to $500 a time." (Rural)

Financial constraints were mentioned by both the urban as well as the rural women as being a factor which limited their use of CAM. This urban woman, for instance, suggested,

"It's so expensive. A lot of (older) people just cannot (afford it). I do think the cost is prohibitive and excessive."

Consistent with the difficulties reported in accessing conventional health services, accessing CAM practitioners can also be problematic for older women who may not be able to drive, either because they previously relied on their husband, or because their licence to drive has been revoked. As one rural woman described,

"I have been isolated, you know, if I can't drive that's a problem."

This view was mirrored by an urban woman,

"I no longer drive, you see. I need transport. Of course friends are always willing. But they are quite old like me, you see."

Although urban areas are more likely to be serviced by public transport, the physical deficits associated with ageing, especially those related to mobility limitations, sometimes influenced where the women would access either CAM practitioners or sources of CAM medications.

"I can get down to (shopping centre), so I get my glucosamine and other things there, but the nearest naturopath is two buses away and sometimes I just find that too much." (Urban)

"They made me feel so much better.....I've only gone off simply because it's so much bother trying to get them." (Rural)

As with other health care providers, retaining a rural CAM workforce can also be difficult, for instance,

"...there was a wonderful man up here but he decided to go because his wife had been bitten by so many ticks." (Rural)

"There was a good fellow in (country town) and he left - of course, all the good ones leave, don't they?" (Rural)

Both urban and rural women suggested that finding a practitioner who suited them was sometimes a matter of trial and error. As one urban woman commented,

"He seemed nice enough, but afterwards I thought that's a peculiar way to do any chiropractic treatment. So I didn't go back."(Urban)

The majority of the women interviewed spoke of their satisfaction with supplements such as glucosamine, fish oil and vitamins, although a number of the rural women commented on the difficulty of obtaining some products, for example,

"There's only one chemist in town who stocks olive extract and if he runs out I sometimes have to wait for weeks for a new lot to come in."(Rural)

The rural women were resourceful in utilising a variety of methods to obtain the products that they used, for instance, "*My sister in Vietnam, she sends it to me." *This was not always successful however as evidenced by the following comment, *"but then, oh no, we've ordered them but they haven't come. I kept getting that and so in the end I thought I can't be bothered with it." *Or as one woman from a large regional town commented,

"I've only gone off it simply because it's so much bother trying to get them."

## Discussion

This qualitative study provides an in-depth account of the factors associated with CAM use in older Australian women. Consistent with results reported in earlier studies [11,12,22,23], this study reveals that CAM use among older women was unlikely to be influenced by aspects of conventional health care ("push factors"), but rather was reflective of the personal beliefs of the women and members of their close social networks ("pull factors").

Previously, rural women have been identified as higher users of CAM [3] and this has been attributed to differences in health service availability and accessibility. But what emerged with the benefit of our qualitative methodology was that the presence of health services was unlikely to influence CAM use. In fact, most of the women, both rural and urban, used conventional medicine in conjunction with CAM. Additionally, these older women were happy to disclose their CAM use to their medical practitioners. This is in contrast to other research which has reported that patients may not tell their doctors that they are using CAM [24-26]. Reasons for not discussing CAM use in previous studies included the belief that the medical practitioner did not need to know, the medical practitioner did not ask about CAM use and a perception that the doctor would not understand or sympathise. However, none of these was cited by the women we interviewed, who appeared to enjoy relationships with their doctors that were characterised by mutual respect and open communications.

Older people may be more reliant on conventional medicine to manage the conditions associated with ageing. All of the women we interviewed reported one or more chronic conditions, with hypertension the most prevalent. Optimal management of this condition relies on pharmaceutical interventions [27] however appropriately managed nutrition is an important adjunct to antihypertensives. During interviews, many of the women emphasised the importance of vitamins and dietary supplements to their well-being.

Members of rural communities share beliefs and experiences that affect their willingness to use different forms of health care. For example, Williams [28] has suggested that a strong sense of place has the potential to enhance the healing process. It is not surprising then that older adults in rural areas may be influenced by the environment and culture of their surrounds and perhaps the close association of rural people with the land will shape and enhance this sense of psychological rootedness. Both CAM and conventional health care services in rural settings are suggested to benefit from robust informal community networks and open patterns of communication among rural practitioners (13,19). Despite this, none of the rural women described themselves, or their reasons for using CAM, as being driven by a sense of geographical place. Rather, both rural and urban women were eloquent in speaking of the importance of self-care and self-reliance in maintaining health and well-being.

The women clearly identified their use of CAM as being driven by personal choice and a desire to maintain control of their healthcare options. These findings are consistent with those reported in other studies [29,30] which suggest that CAM use presents a focus for maintaining current health status as well as providing control in managing health complaints.

Both the urban and the rural women described similar sources of information about CAM, with both groups tending to rely most heavily on advice and information from family and friends. Personal referral and recommendation for CAM has been previously identified as a powerful motivator for CAM use [8]. However, women's groups in rural areas tended to be a source of information that was not reflected in a similar pattern for urban women. For many years, rural women's groups in country Australia have provided social support, information and assistance to country women, and their role as providers of healthcare information builds on that tradition.

While it was also apparent that there were differences between the rural and urban women in the types of CAM used, the reasons for this were mainly due to the difficulties inherent in accessing certain types of CAM in rural areas. In particular, retaining a CAM workforce in rural areas presented challenges that were similar to those faced by conventional service providers. Transport was also identified by both urban and rural women as a barrier to CAM use. Although public transport is more readily available in urban areas, the mobility limitations and reduced agility experienced by women in their eighties limits public transport as a viable option. Similarly, there were no differences between urban and rural women when it came to another common barrier - cost of CAM. Most CAM use in Australia requires direct out-of-pocket payments that may not be refundable by health insurance funds and previous research has indicated that the cost of treatment significantly restricts older people's use of CAM services [31,32]. Many of the women in the current study described how they could not afford more frequent treatments.

This study has a number of limitations which should be considered. Selection of the women who received the mailed invitation was based on a convenience sample. Women who lived in more remote locations may have brought different perspectives to the study. Eligibility was determined by a positive response to an earlier survey question about CAM use. Including women who were not CAM users could have elicited information about why they did not use CAM and how this was influenced by where they lived. Future research could consider conducting comparisons between rural and urban CAM users and non-users.

## Conclusions

This paper provides further support of the importance of CAM use in late adulthood and our results emphasise the positive "pull" factors that underlie older people's decisions to use CAM. Importantly, our qualitative data indicate that older Australian women living in both urban and rural areas share similar motivations, experiences and barriers to using CAM. The women interviewed for this study, regardless of place of residence, provided a snapshot of a cohort of resilient women engaged in managing their own well-being.

## Competing interests

The authors declare that they have no competing interests.

## Authors' contributions

DM devised the idea for the study, collected and analysed the data, and prepared the manuscript. All authors were involved in interpreting the results of the analysis and critically reviewed the manuscript. The final version was approved by all authors.

## Pre-publication history

The pre-publication history for this paper can be accessed here:

http://www.biomedcentral.com/1472-6882/12/34/prepub
